# Classification of *Nostoc*-like cyanobacteria isolated from paddy soil into *Aliinostoc*, *Aulosira*, and *Desmonostoc*

**DOI:** 10.3389/fmicb.2025.1581725

**Published:** 2025-05-20

**Authors:** Hang T. L. Pham, Trang T. Ngo, Thang V. Tran, Tuan A. Duong, Long D. Tran, Anh T. T. Tran, Van T. H. Nguyen, Sang V. Nguyen

**Affiliations:** ^1^HUS High School for Gifted Students, University of Science, Vietnam National University, Hanoi, Vietnam; ^2^Center for Life Sciences Research, Faculty of Biology, University of Science, Vietnam National University, Hanoi, Vietnam; ^3^Faculty of Biology, University of Science, Vietnam National University, Hanoi, Vietnam; ^4^Department of Genetics, Forestry and Agricultural Biotechnology Institute, University of Pretoria, Pretoria, South Africa

**Keywords:** *Aliinostoc*, *Aulosira*, *Desmonostoc*, cyanobacteria classification, *Nostoc*-like morphology, paddy soil, polyphasic approach

## Abstract

Accurate identification of cyanobacterial strains is an essential step for subsequent research to be performed on these organisms. The classification of cyanobacteria in Nostocaceae remains a significant challenge due to the lack of reference data for type species and robust morphological characters for each genus. This study aims to classify 38 new isolated *Nostoc*–like strains at the genus level. The relationship between phylogenetic classification and morphological characteristics at the genus level was also investigated. The 16S rRNA gene sequences served as primary data for phylogenetic classification, supporting the designation of 18 isolates into the *Aliinostoc*, 7 isolates into the *Aulosira*, and 13 isolates into the *Desmonostoc*. Subsequently, we used these isolates as living materials to discover the most distinct features at each genus level of *Aliinostoc*, *Aulosira*, and *Desmonostoc*. As a result, the morphological characteristics of the three genera became distinguishable when grown in the BG11_0_ medium. There, the mature vegetative cells of all isolated strains in the *Aliinostoc* were gray or brown, the strains in the *Aulosira* exhibited basal heterocysts at the beginning of cultivation, and the *Desmonostoc* strains showed the appearance of akinetes in the life cycle as an alternative reproduction. All isolated strains exhibited heterocysts, indicating their ability to fix nitrogen and potentially improve nutrient availability in paddy soil, especially in nitrogen-deficient conditions. This study provides a dataset of 16S rRNA gene sequences and morphological characteristics of *Nostoc* morphotypes, contributing to cyanobacterial taxonomy.

## 1 Introduction

*Nostoc*, a type species of Nostocaceae, is one of the most frequently mentioned genera in studies of cyanobacteria since the first report in the 19th century (Saraf et al., 2018). Many *Nostoc* species were viewed as sources of pharmaceutical compounds such as cryptophycins (Golakoti et al., 1995), cyanovirin-N (Boyd et al., 1997), nostopeptins A and B (Okino et al., 1997), tenuecyclamides A-D and borophycin (Banker and Carmeli, 1998), aeruginosin-865 (Kapuscik et al., 2013), nostosins (Liu et al., 2014), and carbamidocyclophanes (Preisitsch et al., 2015). Nevertheless, *Nostoc muscorum* (Elakbawy et al., 2022; Silambarasan et al., 2021) and *Nostoc sphaeroides* (Chen et al., 2021) were reported as biostimulant producers and nitrogen-fixing organisms for plant growth in the natural environment. Although *Nostoc* was considered a rich source of some lead compounds in drug discovery and a good candidate for agriculture (Fidor et al., 2019), the fact remains that many species labeled as *Nostoc* are morphologically similar but are phylogenetically distant from *Nostoc sensu stricto* (Hrouzek et al., 2005; Řehakova et al., 2007; Papaefthimiou et al., 2008; Cai et al., 2018). Therefore, accurate classification based on molecular phylogenetics is a primary and prerequisite step for any application studies of *Nostoc*-like taxa.

The identification of *Nostoc* based on molecular phylogeny started in Rudi et al. (1997), but numerous genetically distinct species were still assigned to *Nostoc*. This misclassification occurred due to the insufficient reference sequences, which resulted in a false-monophyletic cluster among *Nostoc* and close genera, leading to mistakes in nomenclature. Till 2005, several phylogenetic studies showed that the genus *Nostoc* was polyphyletic (Rajaniemi et al., 2005; Hrouzek et al., 2005; Papaefthimiou et al., 2008); from that point, many *Nostoc*-like species were split out of the core *Nostoc* clade (*Nostoc sensu stricto*) and established new genera within Nostocaceae (Řehakova et al., 2007; Hrouzek et al., 2013; Genuário et al., 2015; Bagchi et al., 2017; Hentschke et al., 2017; Cai et al., 2018). With the concept that a genus should be monophyletic (Komárek et al., 2014), the taxonomy of Nostocaceae is currently improving and developing at more than 10 genera (Maltsevav et al., 2022) based on new proposals and reclassification.

Although molecular phylogeny is indeed essential to the identification and classification of *Nostoc*-like species into different genera (Saraf et al., 2018), many studies have considered morphology as key information on the identification of an organism (Rajaniemi et al., 2005) reflecting the evolutionary history of the organism because it represents the expression of the totality of genes in an organism (Mishra et al., 2015). The characteristic features of each genus facilitate the rapid identification of cyanobacteria strains in samples collected from natural environments, helping isolate new strains with potential applications aligned with initial research goals. To date, limited studies have been focused on describing the common features of strains/species within the *Desmonostoc* (Almeida et al., 2023) and other genera in Nostocaceae. With our knowledge, research to bridge the gap in reference sequences and establish morphological boundary points for each genus is crucial to building a comprehensive cyanobacterial systematics. In Vietnam, the *Nostoc* and their neighbor taxa are frequently found in rice fields; there, they play an important role in the agricultural ecosystem (Pham et al., 2017; Mai et al., 2017). Therefore, in this study, we collected cyanobacterial samples from paddy soil and then isolated cyanobacteria strains with *Nostoc*-like morphology, which serve as essential materials for three main objectives: (1) to maintain alive and pure strains for taxonomic research, (2) to classify isolated strains into the genus level based on 16S rRNA gene sequences and phylogenetic placements, and (3) to summarize the common features at the genus level (*Aliinostoc*, *Aulosira*, and *Desmonostoc*) based on observations of filament color, heterocysts, and akinete characteristics throughout the life cycle of all strains within each genus.

## 2 Materials and methods

### 2.1 Sampling sites and cyanobacteria isolation

The cyanobacteria were isolated from paddy soil and riverside soil in Hanoi (21°00′N – 21°14′N latitude and 105°37′E – 105°51′E longitude), Thanhhoa (19°43′N – 20°27′N latitude and 105°12′E – 105°50′E longitude), and Thaibinh (20°20′N latitude and 106°15′E longitude) provinces. At each study site, samples were taken at three different positions in the period of April to September 2019.

The monoclonal cyanobacterial strains from soil samples were isolated following the description in the previous study (Ngo et al., 2022). All isolated strains were deposited in the cyanobacteria collection at the University of Science, Vietnam National University, Hanoi.

### 2.2 Morphological characterization

Each strain grew on Petri plates either with BG11 medium or with BG11_0_ (nitrogen-deficient BG11) medium using the streaking technqiue. All Petri dishes were incubated under a 12:12 light/dark cycle with white fluorescent irradiation (30 μmol.m^−2^.s^−1^) at 25°C. The morphological features were observed under a Zeiss Axioplan II Fluorescence Microscope with a digital Camera BUC5F-2000C. Images were taken from the colonies of each strain every 3 days throughout their life cycle within 10 weeks. The key diagnostic characteristics, including color and filament shape, the appearance of heterocysts, akinetes, homogonia, and their cell dimensions, were recorded from live materials as described by Pham et al. (2017).

### 2.3 DNA extraction, PCR amplification, and sequencing

For amplifying the 16S rRNA gene, we used three primers that were developed by Boyer et al. (2002):

Primer 1: 5′-CTC TGT GTG CCT AGG TAT CC-3′
Primer 2: 5′-GGG GGA TTT TCC GCA ATG GG-3′
Primer 6: 5′-GAC GGG CCG GTG TGT ACA-3′

Three primers were used for two PCRs. The first round to amplify the partial 16S rRNA sequence and the ITS region (with forward primer 2 and reverse primer 1). Then, the product was reamplified in the second round using forward primer 2 and reverse primer 6 to target only the 16S rRNA gene sequence.

All the procedures for DNA extraction, PCR amplification, and electrophoresis of PCR products followed the description of a previous study (Ngo et al., 2022). All the amplicon products of 38 isolated strains were sent to first BASE (Malaysia) for Sanger sequencing.

### 2.4 Phylogenetic analyses

BLAST searches were carried out using all of the 16S rRNA sequences generated against the NCBI core nucleotide database. Sequences with 98.67% [the species threshold proposed by Kim et al. (2014)] similarity to the query sequences were downloaded and included in a preliminary analysis. The assembled dataset was aligned using MAFFT v7 (Katoh and Standley, 2013) with default settings and trimmed with trimAl with the “-automated1” option activated. The trimmed dataset was then subjected to maximum likelihood (ML) using FastTree 2 (Price et al., 2010), and support values at nodes were based on the Shimodaira–Hasegawa test (Shimodaira and Hasegawa, 1999). Based on the phylogenetic tree obtained with FastTree, three separate datasets were compiled for isolates residing in *Aliinostoc, Aulosira*, and *Desmonostoc*, including all available sequences of type species in each of these genera, together with appropriate outgroup taxa. These sub-datasets were aligned using MAFFT and trimmed using trimAl, as mentioned above. The trimmed datasets were subjected to ML analysis using IQ-TREE 2 (Bui et al., 2020) with the optimal substitution model automatically determined and Bayesian Inference (BI) using MrBayes v3.2.7 (Ronquist et al., 2012) with the same model parameters as determined by IQ-TREE. Run parameters for both ML and BI analyses were the same as those described in Ngo et al. (2022).

## 3 Results

### 3.1 Molecular barcode and phylogeny analysis

Thirty-eight isolated cyanobacteria strains with morphotypes resembling *Nostoc* were used for DNA extraction and amplification of 16S rRNA gene sequences. Then, all 38 16S rRNA gene sequences (about 900 bp in size per sequence) were included along with reference sequences from NCBI databases to construct a phylogeny based on the FastTree 2 tool. As a result, 38 isolated strains in this study were divided into three genera, including *Aliinostoc*, *Aulosira*, and *Desmonostoc,* with 18 strains placed in the *Aliinostoc* genus with a support value of 96.7%, 7 strains grouped in *Aulosira* with a support value of 86.1%, and 13 strains assigned to the *Desmonostoc* genus with a support value of 100% (Supplementary Figure 1). After that, the isolated strains belonging to each genus were further analyzed in a separate phylogenetic tree based on maximum likelihood (ML) and Bayesian Inference (BI) analyses to get a better resolution visualization. We classified one strain into species based on two essential criteria: (1) forming a monophyletic clustering with the type strain and (2) having a 16S rRNA gene sequence identity to the type strain that was more than 98.65% (Kim et al., 2014).

#### 3.1.1 Aliinostoc

**Figure 1:**
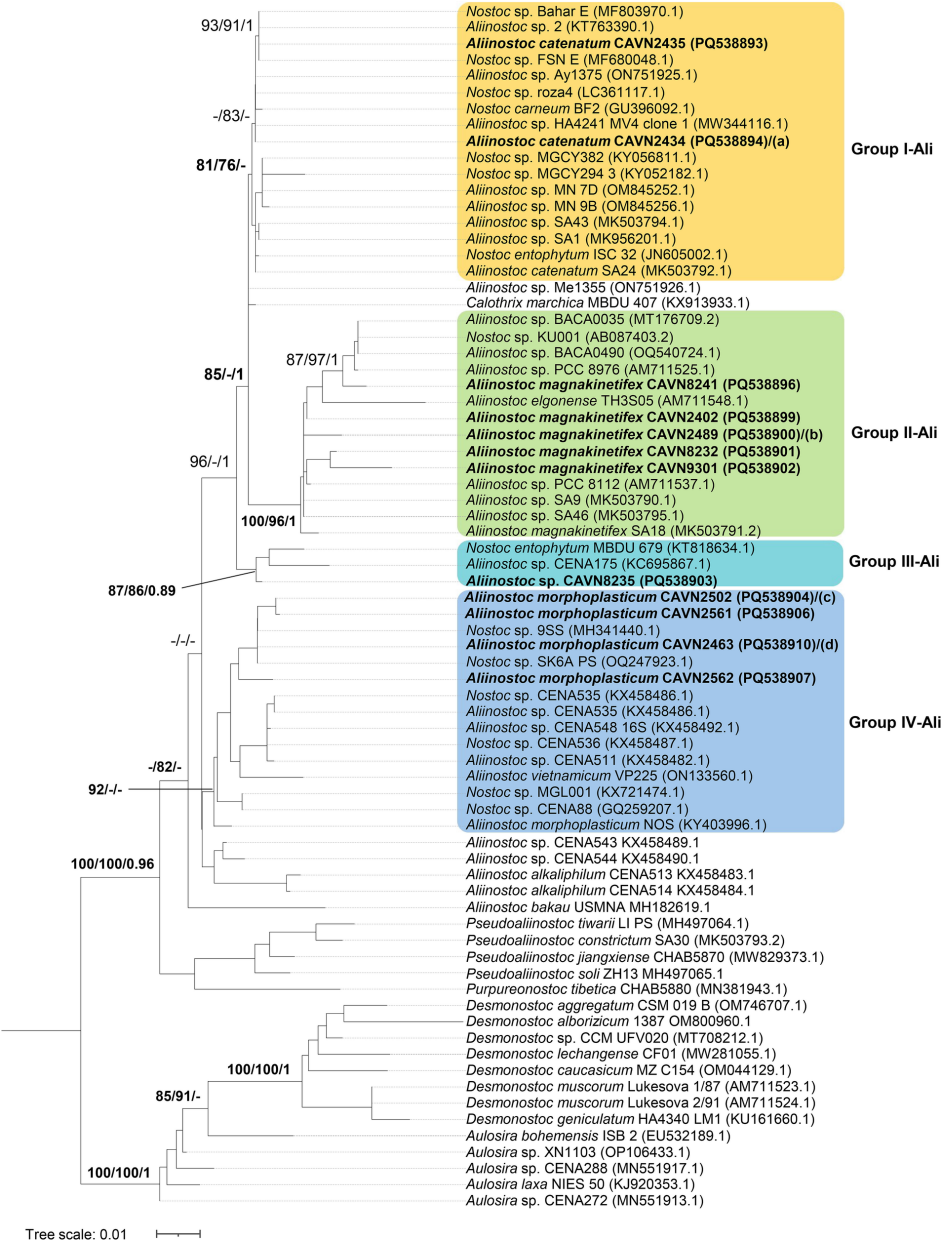
Phylogram derived from maximum likelihood (ML) analysis of 16S rRNA sequence of *Aliinostoc* and selected reference strains from closely related genera outgroup. Branch supports were indicated at nodes, including likelihood ratio test (SH-aLRT ≥ 70%)/ultrafast bootstrap support (BS ≥ 75%)/Bayesian posterior probabilities (BPP ≥ 0.95) greater than 0.95. Scale bar indicates estimated substitutions per site; **(a–d)**: also the positions of *Aliinostoc catenatum* CAVN2436 (PQ538895), *Aliinostoc magnakinetifex* CAVN2437 (PQ538897)/CAVN2438 (PQ538898), *Aliinostoc morphoplasticum* CAVN2501 (PQ538905), and *Aliinostoc morphoplasticum* CAVN2439 (PQ538908)/CAVN2512 (PQ538909), respectively. Strains with 100% 16S rRNA sequence similarity were listed in the same line, separated by “/.”

Eighteen isolated strains in the *Aliinostoc* were divided into four groups corresponding to four species (Figure 1). The group I-Ali, including three strains (CAVN2434, CAVN2435, and CAVN2436), formed a monophyletic group with *Aliinostoc catenatum* SA24 with SH-aLRT/BS/BPP value of 81/76/1. They also showed 99.8%–99.9% similarity of the 16S rRNA gene sequence to *Aliinostoc catenatum* SA24 (Table 1). Therefore, the CAVN2434, CAVN2435, and CAVN2436 strains were assigned to *Aliinostoc catenatum*. Seven strains in group II-Ali (CAVN2402, CAVN2437, CAVN2438, CAVN2489, CAVN8232, CAVN8241, and CAVN9301) were placed into *Aliinostoc magnakinetifex* due to appearing in a monophyletic group along with *A. magnakinetifex* SA18. They shared 98.8%–99.9% of the 16S rRNA gene sequence identity (Table 1) with the strain SA18. The strain CAVN8235 (belonging to group III-Ali) appeared in a monophyletic clade with *Aliinostoc* sp. CENA175 with a strong support of SH-aLRT/BS/BPP (87/86/0.89). However, the two strains only shared 98.5% of 16S rRNA gene sequence similarity (Table 1), indicating that they belonged to different species; then, the strain CAVN8235 was placed in undefined *Aliinostoc* species. The last group of *Aliinostoc* (group IV-Ali) harbored seven strains (CAVN2439, CAVN2463, CAVN2501, CAVN2502, CAVN2512, CAVN2561, and CAVN2562) that formed a monophyletic group along with the type strain *Aliinostoc morphoplasticum* NOS with SH-aLRT/BS/BPP value of 92/−/−. In addition, these strains and *A. morphoplasticum* NOS had 98.8%–99.0% identity (Table 1) of the 16S rRNA gene sequence, indicating that they belonged to *Aliinostoc morphoplasticum* species. Taken together, 18 isolates of *Aliinostoc* were classified into *Aliinostoc catenatum* (3 isolates), *Aliinostoc magnakinetifex* (7 isolates), *Aliinostoc morphoplasticum* (7 isolates), and *Aliinostoc* sp. (1 isolate).

**Table 1:** Percentage identities of the 16S rRNA gene sequence between 18 isolated strains and other most related strains from GenBank in the *Aliinostoc.*

Strains		1	2	3	4	5	6	7	8	9	10	11	12	13	14	15	16	17	18	19
1	CAVN2463/CAVN2439/CAVN2512																			
2	CAVN2562	99.6																		
3	*Aliinostoc morphoplasticum* NOS (KY403996.1)	**98.9**	**99.0**																	
4	*Aliinostoc catenatum* SA24 (MK503792.1)	98.0	98.3	98.3																
5	CAVN2434/CAVN2436	98.1	98.5	98.5	**99.9**															
6	CAVN2435	98.2	98.6	98.6	**99.8**	99.9														
7	CAVN8241	97.4	97.5	97.7	98.7	98.8	98.7													
8	*Nostoc entophytum* MBDU679 (KT818634.1)	98.6	98.2	98.0	98.9	98.8	98.7	98.6												
9	CAVN8235	98.5	98.1	97.9	98.8	98.7	98.6	98.5	99.9											
10	CAVN2402	97.9	98	98.2	98.7	98.8	98.9	98.8	98.1	98.0										
11	CAVN2489/CAVN2437/CAVN2438	97.9	98	98.2	98.7	98.8	98.9	98.8	98.1	98.0	100									
12	CAVN8232	98.0	98.1	98.1	98.6	98.7	98.8	98.7	98.0	97.9	99.9	99.9								
13	*Aliinostoc magnakinetifex* SA18 (MK503791.2)	97.9	98	98.2	98.7	98.8	98.9	**98.8**	98.1	98.0	**99.9**	**99.9**	**99.9**							
14	CAVN2501/CAVN2502	100	99.6	**98.9**	98	98.1	98.2	97.4	98.6	98.5	97.9	97.9	98.0	97.9						
15	CAVN2561	99.9	99.5	**98.8**	97.9	98.0	98.1	97.3	98.5	98.3	97.7	97.7	97.9	97.7	99.9					
16	*Aliinostoc vietnamicum* VP225 (ON133560.1)	98.1	98.0	98.0	98.9	98.8	98.9	97.9	98.6	98.5	98.3	98.3	98.5	98.3	98.1	98.0				
17	CAVN9301	97.9	98.0	98.2	98.7	98.8	98.9	98.8	98.1	98.0	100	100	99.9	**99.9**	97.9	97.7	98.3			
18	*Aliinostoc* sp. CENA175 (KC695867.1)	97.4	97.5	97.0	98.5	98.3	98.2	97.6	98.6	**98.5**	97.4	97.4	97.3	97.4	97.4	97.3	97.6	97.4		
19	*Aliinostoc elgonense* TH3S05 (AM711548.1)	97.5	97.6	97.4	97.1	97.3	97.4	96.8	96.6	96.4	97.3	97.3	97.4	97.3	97.5	97.4	97.0	97.3	95.7	

Numbers in bold are the highest identity values of 16S rRNA gene sequence between isolates and referent strains. Strains with 100% 16S rRNA sequence similarity were listed in the same line separated by “/.”

#### 3.1.2 Aulosira

**Figure 2:**
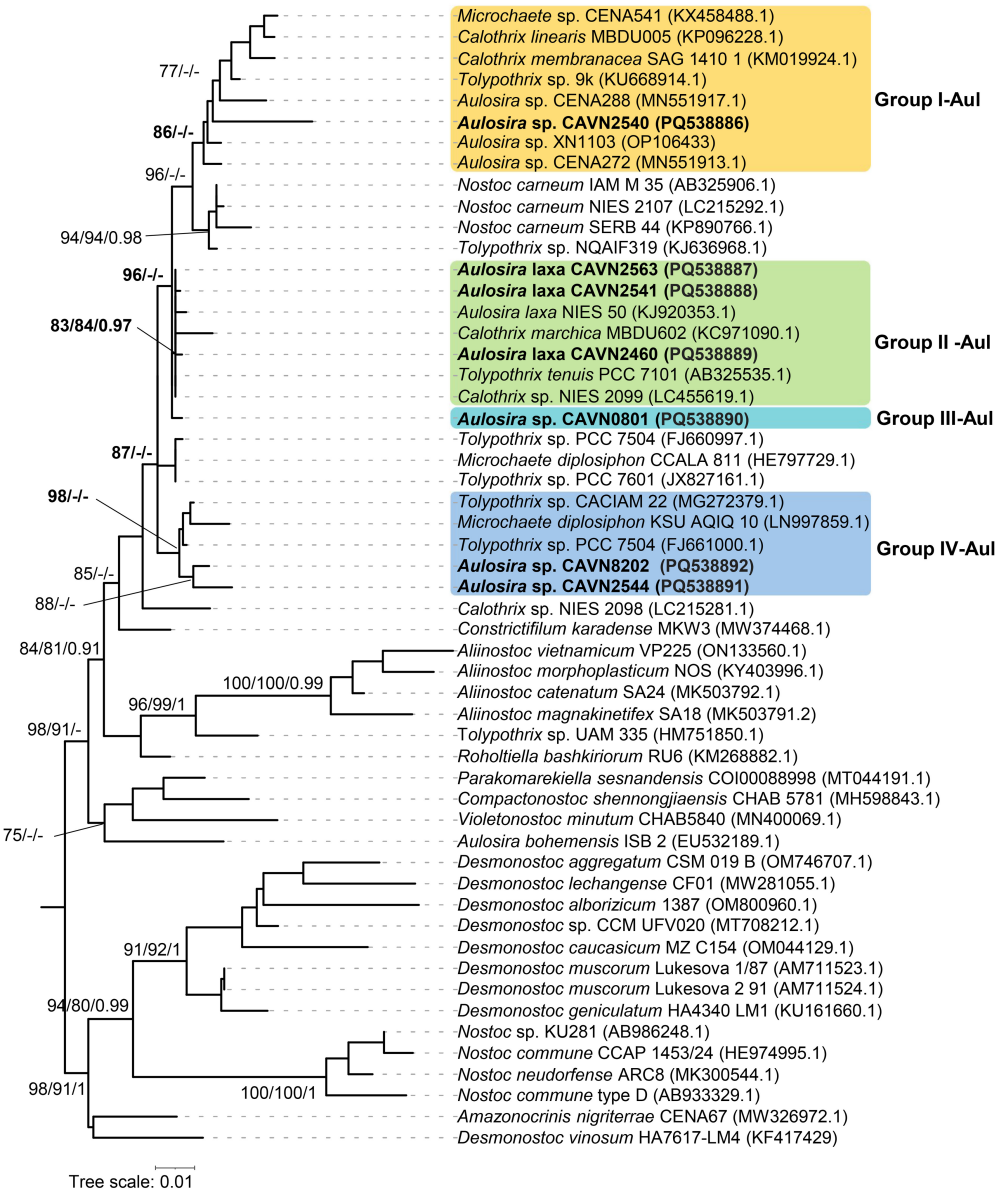
Phylogram derived from maximum likelihood (ML) analysis of 16S rRNA sequence of *Aulosira* and selected reference strains from closely related genera outgroup. Branch supports were indicated at nodes, including likelihood ratio test (SH-aLRT ≥ 70%)/ultrafast bootstrap support (BS ≥ 75%)/Bayesian posterior probabilities (BPP ≥ 0.95) greater than 0.95. Scale bar indicates estimated substitutions per site.

Seven isolated strains appeared in the *Aulosira* cluster and were divided into four groups based on the phylogenetic placement (Figure 2) and 16S rRNA gene sequence identities to the *Aulosira* type strain, *Aulosira laxa* NIES-50 (Table 2). The strain CAVN2540 appeared in group I-Aul along with *Aulosira* sp. CENA288, *Aulosira* sp. CENA272, and *Aulosira* sp. XN1103. This group was more supported by ML rather than BI analyses, with an SH-aLRT of 86%. In addition, the high identity of 99.2%–99.6% in the 16S rRNA gene sequence between CAVN2540 and the three reference strains indicated that the four strains belonged to the same species. Group II-Aul included the CAVN2460, CAVN2541, and CAVN2563 strains, which had more than 99.7% identity of the 16S rRNA gene sequences to that of *Aulosira laxa* NIES-50. The monophyletic branch of these three strains to *Aulosira laxa* NIES-50 in the phylogenetic tree with SH-aLRT/BS/BPP values of 83/84/0.97 indicated that they can be placed into *Aulosira laxa*. Group III-Aul contained only one strain, CAVN0801. Although it shared 99.6% similarity in the 16S rRNA gene sequence to the *Aulosira laxa* NIES-50, the separated position apart from *Aulosira laxa* NIES-50 indicated that strain CAVN0801 belonged to a sister species of *Aulosira laxa*. Similarly, the strains CAVN2544 and CAVN8202 in group IV-Aul were not identified at the species level due to a lack of referent species; thus, they were classified into *Aulosira* sp. In short, among seven strains in the *Aulosira* cluster, three strains (CAVN2460, CAVN2541, and CAVN2563) were assigned to *Aulosira laxa*, and four left strains belonged to three undefined species of *Aulosira*.

**Table 2:** Percentage identities of the 16S rRNA gene sequence between seven isolated strains and other most related strains from GenBank in the *Aulosira.*

Strains		1	2	3	4	5	6	7	8	9	10	11	12	13	14	15	16
1	CAVN2563																
2	CAVN2541	100															
3	CAVN0801	99.8	99.8														
4	CAVN2460	99.9	99.9	99.7													
5	CAVN8202	99.2	99.2	99.3	99.1												
6	CAVN2544	99.0	99.0	99.2	98.9	99.2											
7	CAVN2540	99.0	99.0	99.0	99.1	98.7	98.7										
8	*Nostoc carneum* IAM M35 (AB325906.1)	99.0	99.0	99.0	99.1	98.7	98.8	**99.8**									
9	*Aulosira* sp. CENA272 (MN551913.1)	99.1	99.1	99.1	99.0	99.0	99.1	**99.2**	99.4								
10	*Aulosira* sp. XN1103 (OP106433)	99.0	99.0	99.2	98.9	98.5	98.8	**99.6**	99.6	99.4							
11	*Aulosira laxa* NIES 50 (KJ920353.1)	**99.8**	**99.8**	**99.6**	**99.7**	**99.0**	**98.8**	**98.8**	98.8	98.9	98.8						
12	*Tolypothrix* sp. CACIAM 22 (MG272379.1)	99.4	99.4	99.4	99.6	98.9	98.7	98.9	98.9	98.8	98.7	99.2					
13	*Aulosira* sp. CENA288 (MN551917.1)	98.9	98.9	98.9	98.8	98.5	98.8	**99.2**	99.1	98.8	99.1	98.7	98.5				
14	*Calothrix membranacea* SAG 1410 1 (KM019924.1)	98.5	98.5	98.5	98.7	98.0	98.0	98.9	98.7	98.3	98.7	98.3	98.9	98.5			
15	*Microchaete diplosiphon* KSU AQIQ 10 (LN997859.1)	98.8	98.8	98.8	98.9	98.4	98.2	98.4	98.4	98.1	98.0	98.5	99.1	98.1	98.4		
16	*Aulosira bohemensis* ISB 2 (EU532189.1)	97.4	97.4	97.6	97.3	97.2	96.9	97.5	97.3	97.2	97.8	97.2	97.8	97.2	97.3	97.1	

Number in bold are the highest identity values of 16S rRNA gene sequence between isolates and referent strains.

#### 3.1.3 Desmonostoc

**Figure 3:**
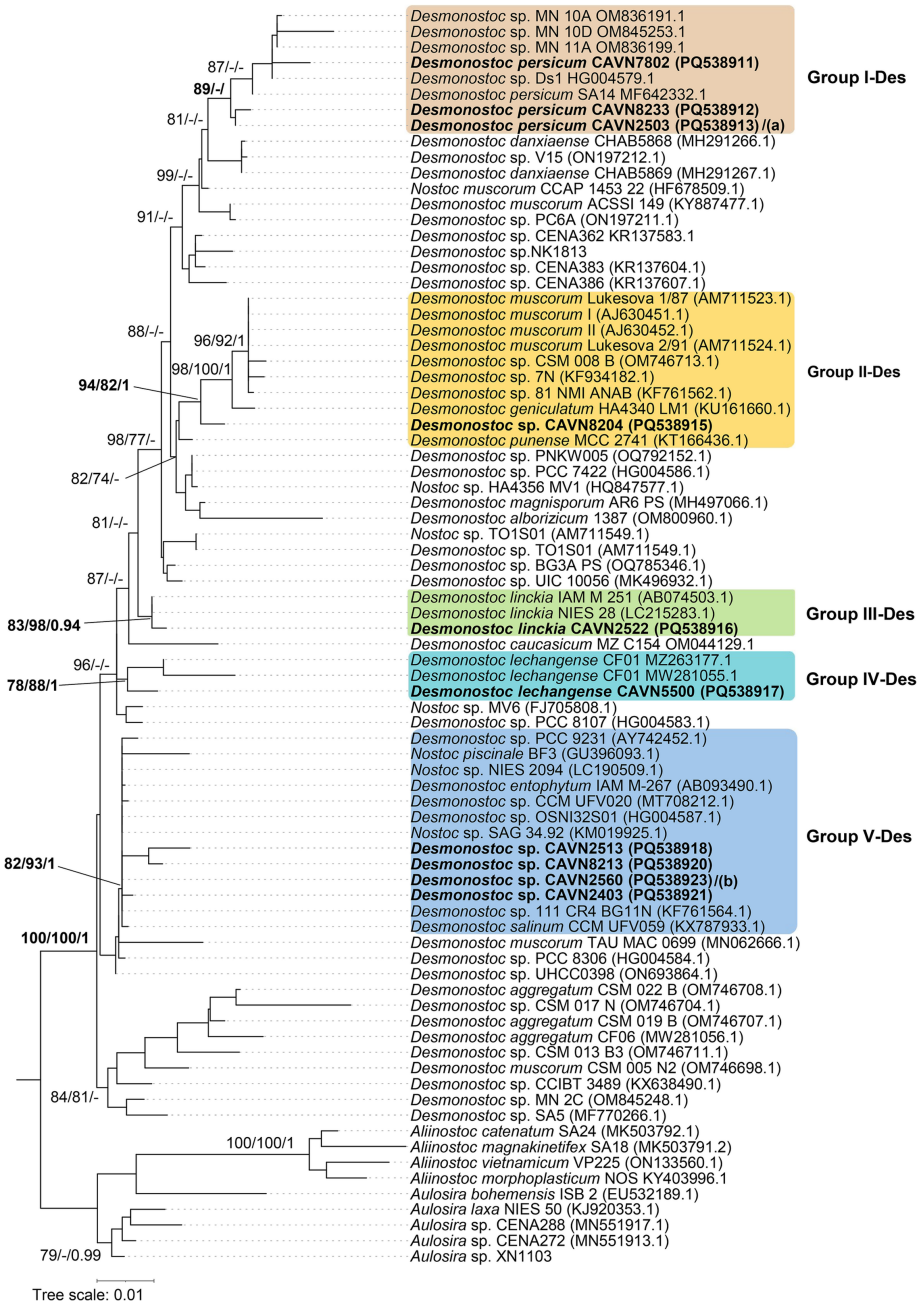
Phylogram derived from maximum likelihood (ML) analysis of 16S rRNA sequence of *Desmonostoc* and selected reference strains from closely related genera outgroup. Branch supports were indicated at nodes, including likelihood ratio test (SH-aLRT ≥ 70%) / ultrafast bootstrap support (BS ≥ 75%)/Bayesian posterior probabilities (BPP ≥ 0.95) greater than 0.95. Scale bar indicates estimated substitutions per site; Panels **(a,b)** also the positions of *Desmonostoc persicum* CAVN2441 (PQ538914), and *Desmonostoc* sp. CAVN6515 (PQ538919)/CAVN2525 (PQ538922), respectively. Strains with 100% 16S rRNA sequence similarity were listed in the same line, separated by “/.”

Thirteen isolated strains in *Desmonostoc* formed a monophyletic cluster with the core *Desmonostoc* strains by a maximum SH-aLRT/BS/BPP value of 100/100/1. They were separated into five groups corresponding to five species (Figure 3). The group I-Des, including CAVN2441, CAVN2503, CAVN7802, and CAVN8233, was assigned to *Desmonostoc persicum* based on close distance in the phylogenetic tree and shared 99.3% 16S rRNA gene sequence similarity (Table 3) to the strain *Desmonostoc persicum* SA14. The strain CAVN8204 (in group II-Des) was placed in the same clade as *Desmonostoc muscorum* 1/87, *Desmonostoc geniculatum* HA4340 LM1, and *Desmonostoc punense* MCC 2741. The highest identity value (99.2%) of 16S rRNA gene sequences was found between CAVN8204 and *Desmonostoc punense* MCC 2741. Still, the phylogenetic topology, either by ML or BI analysis, was not supported for assigning CAVN8204 and *Desmonostoc punense* MCC 2741 into one species. Therefore, the strain CAVN8204 was defined as a member of an undefined *Desmonostoc* species. The strain CAVN2522 (of the group III-Des) formed a monophyletic cluster with *Desmonostoc linckia* IAM M-251 and *Desmonostoc linckia* NIES 28, with the SH-aLRT/BS/BPP values of 83/98/0.94. Nevertheless, it shared 99.8% 16S rRNA gene sequence similarity to *Desmonostoc linckia* IAM M-251 (Table 3); thus, the strain CAVN2522 was classified as *Desmonostoc linckia*. The strain CAVN5500 (of group IV-Des) appeared in the same node as the referent strain of *Desmonostoc lechangense* CF01. The monophyletic evolution and the 99.8% 16S rRNA gene sequence similarity to *Desmonostoc lechangense* CF01 indicated that the strain CAVN5500 was a member of *Desmonostoc lechangense*. Group V-Des, including CAVN2403, CAVN2513, CAVN2525, CAVN2560, CAVN6515, and CAVN8213, was plotted within a cluster with *Desmonostoc entophytum* IAM M-267 and *Desmonostoc salinum* CCM UFV059 with a strong SH-aLRT/BS/BPP value of 82/93/1. However, based on the ecological aspect, these strains threaten the paddy soil rather than the rock surface or saline-alkaline environment, as in the case of *Desmonostoc entophytum* IAM M-267 and *Desmonostoc saline* CCM UFV059 (de Alvarenga et al., 2018; Almeida et al., 2023). Thus, these six isolated strains were designated as *Desmonostoc* spp. Briefly, 13 isolates of *Desmonostoc* were classified into *Desmonostoc persicum* (4 isolates), *Desmonostoc linckia* (1 isolate), *Desmonostoc lechangense* (1 isolate), and two unidentified *Desmonostoc* spp. (7 isolates).

**Table 3:** Percentage identities of the 16S rRNA gene sequence between 13 isolated strains and other most related strains from GenBank in the *Desmonostoc.*

Strains		1	2	3	4	5	6	7	8	9	10	11	12	13	14	15	16	17	18	19
1	*Desmonostoc salinum* CCM UFV059 (KX787933.1)																			
2	CAVN2560/CAVN2525/CAVN6515	**99.8**																		
3	CAVN2403	**99.8**	100																	
4	*Nostoc entophytum* IAM M 267 (AB093490.1)	99.6	**99.9**	**99.9**																
5	CAVN5500	99.3	99.5	99.5	99.4															
6	*Desmonostoc lechangense* CF01 (MZ263177.1)	99.0	99.3	99.3	99.2	**99.8**														
7	*Nostoc linckia* IAM M 251 (AB074503.1)	98.6	98.8	98.8	98.7	99.0	98.8													
8	CAVN2522	98.3	98.6	98.6	98.4	98.8	98.6	**99.8**												
9	*Desmonostoc* sp. NK1813	98.6	98.8	98.8	98.7	98.3	98.1	98.6	98.3											
10	CAVN8233	98.8	99.0	99.0	98.9	99.0	99.0	99.0	98.8	99.0										
11	CAVN2503/CAVN2441	98.8	99.0	99.0	98.9	99.0	99.0	99.0	98.8	99.0	100									
12	*Desmonostoc persicum* SA14 (MF642332.1)	98.8	99.0	99.0	98.9	99.0	99.0	99.0	98.8	99.0	100	100								
13	CAVN7802	98.4	98.7	98.7	98.6	98.7	98.7	98.9	98.7	99.2	**99.3**	**99.3**	**99.3**							
14	CAVN8204	97.7	98.0	98.0	97.8	98.2	98.0	98.7	98.4	98.3	98.4	98.4	98.4	98.4						
15	*Desmonostoc muscorum* Lukesova 1/87 (AM711523.1)	97.5	97.7	97.7	97.6	97.7	97.5	98.4	98.2	97.8	97.7	97.7	97.7	98.0	98.6					
16	CAVN2513/CAVN8213	99.6	99.9	99.9	**99.8**	99.4	99.2	98.7	98.4	98.7	98.9	98.9	98.9	98.6	97.8	97.6				
17	*Desmonostoc punense* MCC2741 (KT166436.1)	97.8	98.1	98.1	98.0	98.3	98.1	98.8	98.6	98.4	98.6	98.6	98.6	98.6	**99.2**	98.0	98.0			
18	*Desmonostoc geniculatum* HA4340 LM1 (KU161660.1)	96.9	97.1	97.1	97.0	97.1	96.9	97.6	97.4	97.5	97.4	97.4	97.4	97.4	98.7	99.2	97.0	98.1		
19	*Nostoc piscinale* BF3 (GU396093.1)	99.4	99.6	99.6	99.5	99.2	98.9	98.7	98.4	98.6	98.7	98.7	98.7	98.4	97.8	97.6	99.5	98.0	97.0	

Number in bold are the highest identity values of 16S rRNA gene sequence between isolates and referent strains. Strains with 100% 16S rRNA sequence similarity were listed in the same line separated by “/.”

### 3.2 Morphology characteristics analysis at the genus level

The isolated strains within each genus were carefully observed throughout the life cycle, growing on agar plates on BG11 and BG11_0_ media. All isolated strains exhibited heterocysts (Supplementary Table 1) when cultured in nitrogen-deficient BG11 medium (BG11_0_), indicating their ability to fix nitrogen. The features of all strains belonging to one genus were gathered to identify signature characteristics at the genus level.

#### 3.2.1 *Aliinostoc* genus

**Figure 4:**
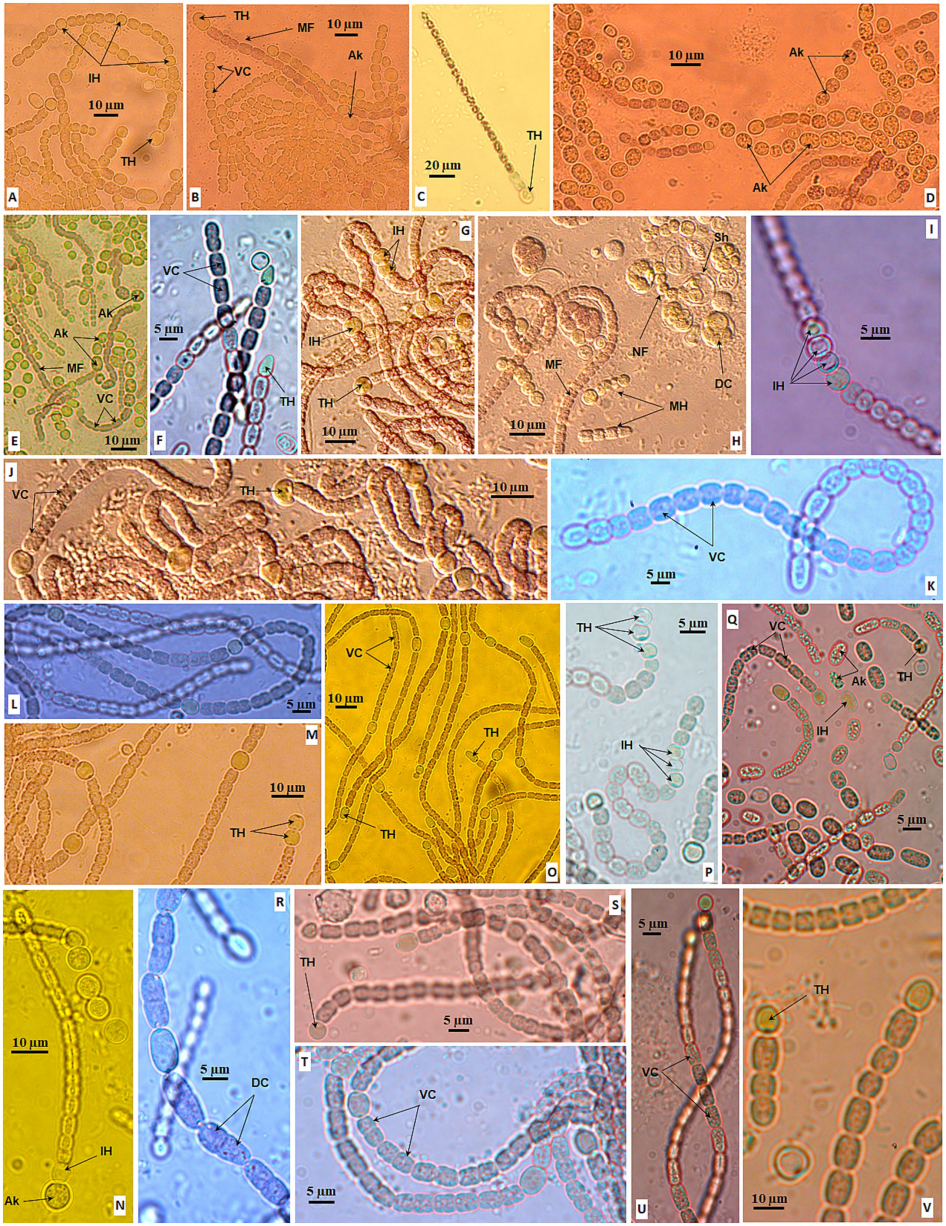
Morphology of strains in *Aliinostoc* Strain CAVN2562 **(A,B)**, CAVN2501 **(C)**, CAVN2561 **(D)**, CAVN2502 **(E)**, CAVN8235 **(F)**, CAVN2438 **(G–I)**; CAVN2437 **(J)**, CAVN2489 **(K)**, CAVN2402 **(L)**, CAVN2463 **(M,N)**, CAVN2435 **(O)**, CAVN9301 **(P)**, CAVN2436 **(Q)**, CAVN2512 **(R)**, CAVN2439 **(S)**, CAVN8232 **(T)**, CAVN2434 **(U)**, and CAVN8241 **(V)**. Ak, akinete; DC, dividing cell; IH, intercalary heterocyte; MH, motile hormogonia; NF, new filament; MF, mature filament; Sh, sheath; TC, terminal cell; TH, terminal heterocyte; VC, vegetative cell.

All 18 isolated strains in the *Aliinostoc* genus were light brown to gray (Figure 4). There was no notable morphological difference among the strains when grown in the BG11_0_ medium or the BG11 medium. However, the strains in the *Aliinostoc* cluster were variable in the features of cells and filaments. The filaments existed in short, straight forms (Figures 4C,F) or long-coiled forms (Figures 4G,J,L). Besides the motile hormogonia, the new filament might develop from one light green–brown cell after several divisions inside a spherical sheath. Then, it was released as a straight filament (Figure 4H). The vegetative cells in the filament were nearly uniform and tightly attached (Figures 4B,K,O). They were variable, from barrel-shaped (Figures 4K,T), cylindrical (Figures 4F,U), or disc-shaped (Figure 4J) with clearly constricted walls, divided in the perpendicular plane (Figure 4R). The mature (old) vegetative cells became brownish-gray (Figures 4B,E,H). The heterocysts occurred both at the terminal and middle of the filament (Figures 4A,G). The intercalary heterocysts appeared in a pattern separated by five vegetative cells (Figure 4A) or in a series of one to four (Figures 4G,I,P). The terminal heterocysts were diverse, from semi-round (Figure 4J) to round (Figures 4M,S), oval (Figure 4B), or pointed (Figures 4F,O); appeared in two (Figure 4M) or three (Figures 4C,P). The terminal heterocyst might appear in the very short filament of four cells (Figure 4V). The akinetes were round or oval and loosely appeared in series (Figures 4D,Q) at the beginning or middle of the filament (Figure 4E). The akinetes formed between two heterocysts (Figure 4Q) or lay next to heterocysts (Figure 4N). The akinetes contained many blue-green granular that made the chains brighter than the chains of vegetative cells (Figure 4E).

#### 3.2.2 *Aulosira* genus

**Figure 5:**
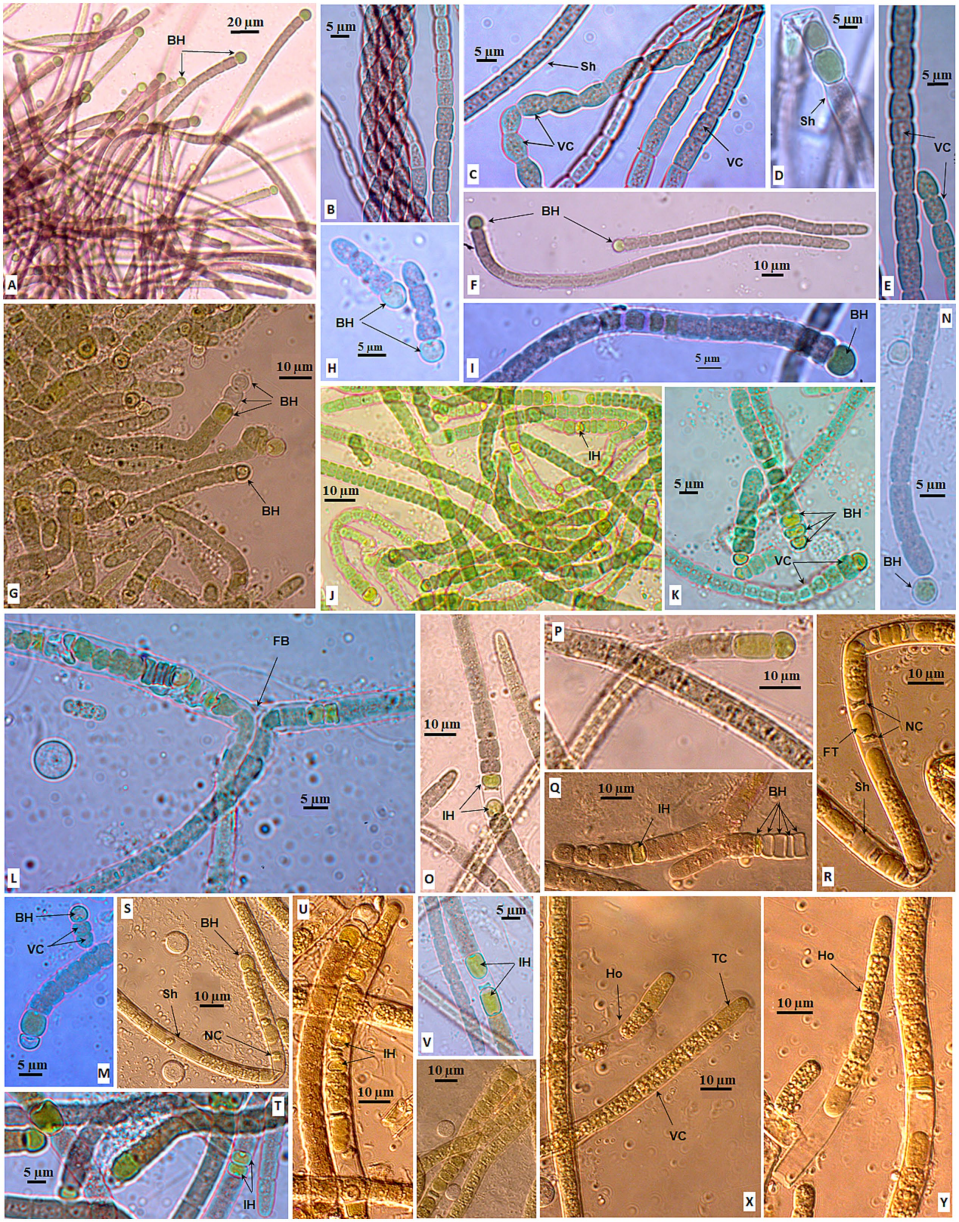
Morphology of strains in *Aulosira.* Strain CAVN2563 **(A–F)**, CAVN2544 **(G)**, CAVN0801 **(H,I)**, CAVN8202 **(J–M)**, CAVN2541 **(N)**, CAVN2540 **(O,P)**, and CAVN2460 **(Q–Y)**. BH, basal heterocyte; FB, false branching; FT, fertile trichome; Ho, hormogonia; IH, intercalary heterocyte; NC, necrotic cell; Sh, sheath; TC, terminal cell; VC, vegetative cell.

Seven isolated strains of the *Aulosira* genus were in gray (Figures 5A,B), olive green (Figure 5G) or blue-green (Figures 5J–K). When growing in the BG11 medium, strains of this genus showed the *Nostoc*-resembled morphology by long filaments of cylindrical-, oval-, or barrel-shaped vegetative cells (Figures 5C,E) with obvious cross walls in fine hyaline sheaths (Figure 5C) or thick sheaths (Figure 5D). Sometimes, the filament was very long with a diffusion sheath, 2–3 filaments twisted together (Figure 5B). The basal heterocysts rarely appeared (Figure 5I); even so, they detached from the filament after 4 weeks (Figure 5N).

When growing in the BG11_0_ agar medium, all isolated strains of this genus shared one common characteristic that did not appear in *Nostoc* strains. Almost all filaments were heteropolar, including one pale-green basal heterocyst, with a minimum of two vegetative cells at the first week of cultivation (Figures 5F–H,M). The basal heterocysts existed on the filament from the young to the mature and old phases. At the mature phase, one to five basal heterocysts (Figures 5G,K,P,Q) might have appeared successively in the filaments; one to three cells next to the vegetative cells remained green, and the terminal cells might be necrotic. On the other hand, strains exhibited diverse morphology in the BG11_0_ agar medium. The basal heterocyst was round (Figure 5F), oblong (Figure 5P), or disc-shaped (Figures 5K,Q). At the mature phase, the heterocyst also appeared intercalar single (Figures 5J,Q) or in pairs (Figure 5T) or separated by one or two necrotic vegetative cells (Figures 5O,U,V). False branching was rarely observed with or without heterocysts at branching (Figure 5L).

The vegetative cells with prominent granules (Figure 5X) were similar to those when growing in BG11, with clear constrictions at the cross walls in the young filaments (Figures 5C,E) and slight constrictions at the cross walls in the mature filaments (Figure 5N); sometimes these cells swelled in the middle or basal of the filament (Figure 5K). The filament width ranged from 4.0 μm to 11.4 μm. The terminal cells were attenuated or conical, sometimes increasing twice the length of the adjacent vegetative cell (Figure 5X). The sheath was not visible in the first week of cultivation (Figure 5H) but was thick or thin in the following weeks (Figures 5I,P,R,S). Many vegetative cells were necrotic at week eight, but the heterocyst was still retained in the basal filament (Figure 5S). For reproduction, short fertile trichomes (Figures 5R,W) included round or oval cells (spores) that gradually formed between necrotic vegetative cells and were released from old filaments as hormogonia (Figures 5X,Y). The akinetes were not observed at all in isolated *Aulosira* strains in BG11_0_ and BG11 media.

#### 3.2.3 *Desmonostoc* genus

**Figure 6:**
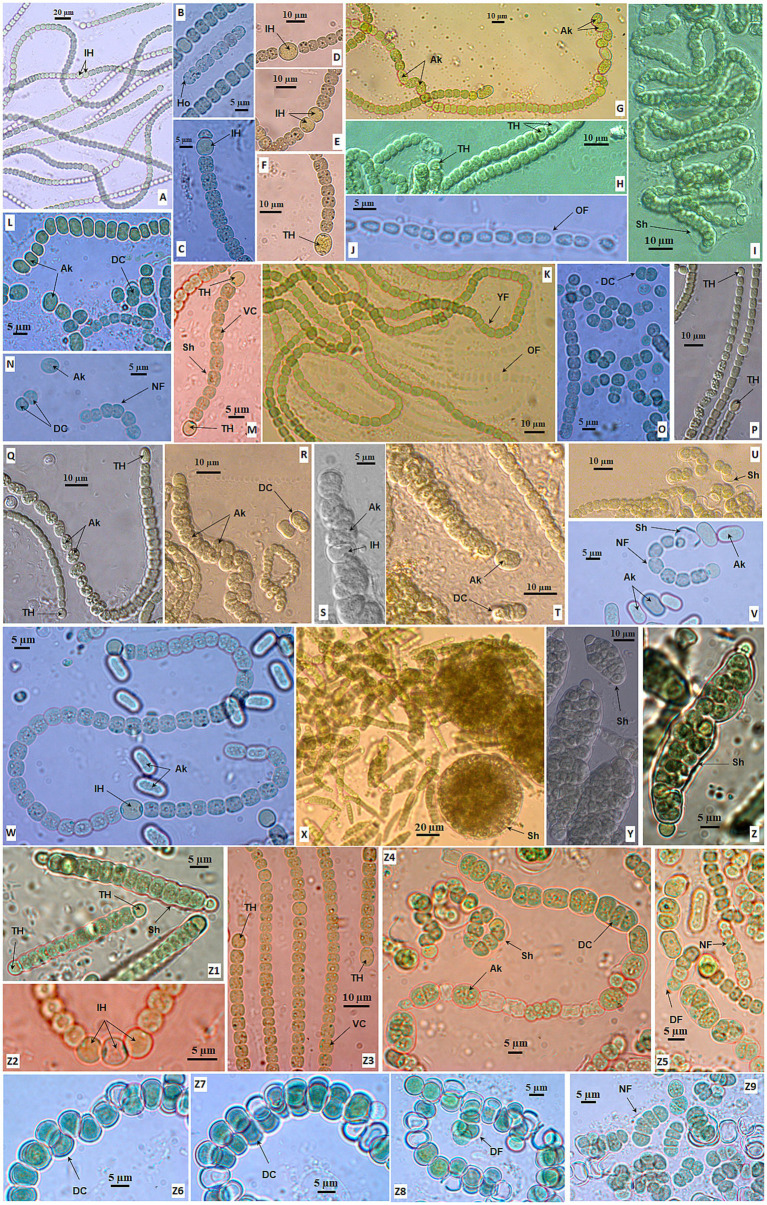
Morphology of strains in *Desmonostoc* CAVN2503 **(A,B)**, CAVN2441 **(C–F)**, CAVN8233 **(G)**, CAVN2525 **(H,I)**, CAVN2560 **(J,K)**, CAVN6515 **(L,M)**, CAVN8213 **(N,O)**, CAVN2513 **(P,Q)**, CAVN2403 **(R–U)**, CAVN8204 **(V–W)**, CAVN2522 **(X–Z1)**, CAVN5500 **(Z2–Z5)**, and CAVN7802 **(Z6–Z9)**. Ak, akinete; DC, dividing cell; DF, developing filaments; Ho, hormogonia; IH, intercalary heterocyte; NF, new filament; OF, old filament; Sh, sheath; TH, terminal heterocyte; VC, vegetative cell.

**Figure 7:**
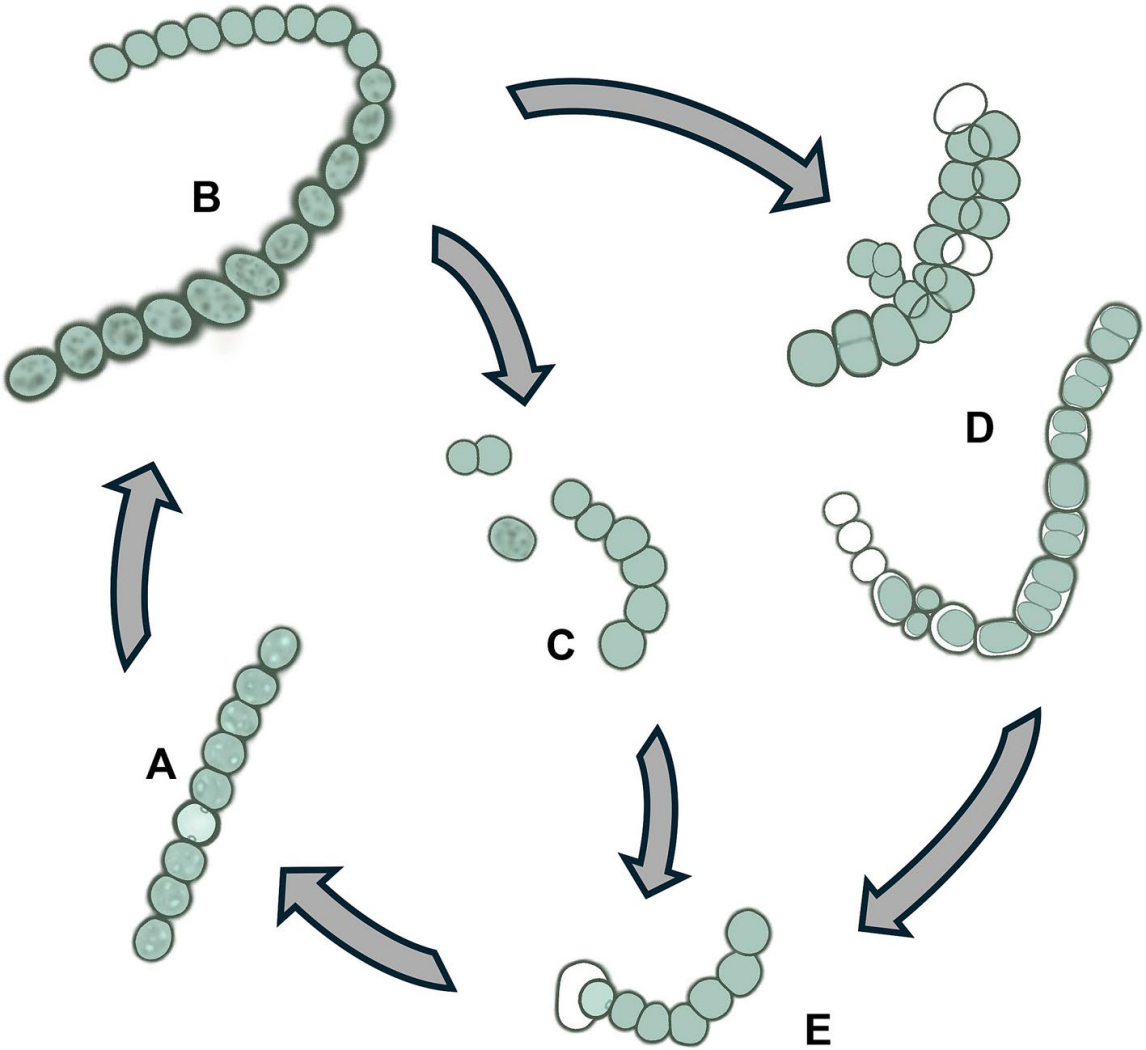
Life cycle of *Desmonostoc* **(A)** Young filament with uniform vegetative cells, **(B)** mature filament with akinetes, **(C)** akinetes germination outside the filament, **(D)** Akinetes germination inside the filament, **(E)** New filament releasing from akinetes sheath.

Thirteen *Desmonostoc* strains were in gray-green (Figures 6A–F), olive-green (Figures 6P,Q), or light blue-green (Figures 6J–L,O,Z4–Z9). The sheath was diffluent (Figures 6I,K), thin (Figure 6M), or enclosing the colony (Figure 6X). The filaments were isopolar, straight (Figures 6A,B,Z3), or coiled (Figure 6I) with uniform and tightly attached vegetative cells in the young filaments, and sometimes they became loosened, dissipated in the old filament (Figures 6J,K). The vegetative cells fall in small size (2.5–3.7 μm in width) (Figures 6K,P) or big (6.3–7.5 μm in width) (Figures 6B,F). The vegetative cells were equally barrel-shaped (Figures 6M,W,Z3) with clearly constricted cell walls. The heterocysts were big and round or oval shape (Figures 6C–F,Q,Z3). The heterocysts (one or two) appeared at terminal filaments (Figures 6F,H,P,Z3), or even at both ends of short filaments (Figures 6M,Z1); or at intercalary positions from one to three cells (Figures 6A,E,W,Z2). The akinetes were oval or cylindrical, much larger than vegetative cells (Figures 6G,L,V,W,Z4). They appeared in series at intercalary (Figure 6P) or terminal (Figure 6G) loosely (Figure 6Q) or tightly linked (Figure 6R), sometimes inserted by heterocysts (Figure 6S). The appearance of akinetes was found in all isolated *Desmonostoc* strains, and they play an important role in reproduction in addition to homogonia (Figure 6B). Akinetes might germinate and develop into new filaments in two strategies. One was that the akinete might detach from the chains (Figures 6T,V,W) and germinate into new filaments (Figures 6L,N,O,R,T). The new filament was released from the sheath of an akinete (Figures 6U,V). The sheath might retain and cover the developing filament (Figures 6Y,Z). Second was that the akinete germinated inside the maternity filament with the perpendicular dividing plane (Figure 6Z4) or parallel dividing plane (Figures 6Z6–Z8) to give rise to the developing filaments (Figures 6Z5,Z8). After that, the new filaments (Figures 6Z5,Z9) were released from the maternity filament. Therefore, the life cycle of *Desmonostoc* could be concluded as illustrated in Figure 7, including (1) young filament with uniform vegetative cells (Figure 7A); (2) mature filament without akinetes (spores) or with akinetes (Figure 7B); (3) akinetes germination outside the filament (Figure 7C) or inside the filament (Figure 7D); and (4) new filament releasing from akinetes sheath (Figure 7E).

#### 3.2.4 Morphological characteristic delimitation at the genus level

The morphological and morphometrical characteristics of the three genera are presented in Table 4. The sizes of vegetative cells, heterocysts, and akinetes were summarized from all isolates within each genus, which are provided in Supplementary Table 1. Three genera share several traits, such as the appearance of heterocysts, especially at the intercalary trichome, and the hormogonia productions. The *Aulosira* and *Desmonostoc* genus had several strains in blue-green color, the typical color of cyanobacteria, due to the occurrence of chlorophyll and phycocyanin pigments. However, all 18 *Aliinostoc* strains exhibited gray or brownish trichomes or colonies, never in blue-green color at vegetative cells. In another aspect, *Aulosira* was distinguished from two other genera by the feature of trichome. All *Aulosira* strains showed the heteropolar trichome with heterocyst in most lifetimes when growing in a solid nitrate-deficient medium. Several strains of *Aliinostoc* also exhibited terminal heterocysts in the young trichome, but more heterocysts then appeared at other terminals of the mature trichome. Unlike that, all *Aulosira* strains in our study never had the heterocyst at apical of trichomes. Although the size of vegetative cells of *Aulosira* was 1.7–2.0-fold greater than *Aliinostoc* and *Desmonostoc*, we did not consider this trait as a standard feature of *Aulosira* due to the variety of cell sizes in cyanobacteria in general. Nevertheless, the akinete chain was observed in all strains of *Desmonostoc*, suggesting that it can be a prominent characteristic at the genus level.

**Table 4:** Morphological comparison at genus level for *Aliinostoc*, *Aulosira*, and *Desmonostoc* in this study.

*Traits*	*Aliinostoc*	*Aulosira*	*Desmonostoc*
Colony color	Light brown or gray	Gray, olive green, or blue-green	Gray-green, olive-green, or light blue-green
Trichome	Isopolar	Heteropolar	Isopolar
Vegetative cells	brown or gray	Gray, olive green, or blue-green	Gray-green, olive-green, or dark blue-green
	2.3–5.4 μm wide	4.0–11.4 μm wide	2.5–7.7 μm wide
	1.7–7.7 μm long	4.1–13.9 μm long	2.4–10.0 μm long
Heterocyst	Terminal or intercalary	Pale-green basal heterocyte intercalary, never in apical	Terminal or intercalary
	2.6–6.7 μm wide	4.4–8.5 μm wide	2.7–10.0 μm wide
	2.4–9.6 μm long	4.5–14.5 μm long	2.7–14.8 μm long
Akinete	Occasionally with blue-green granular Terminal or intercalary	Not observed	Terminal or intercalary
	3.0–6.6 μm wide		3.9–13.8 μm wide
	3.0–11.4 μm long		3.8–13.5 μm long
Reproduction strategy	Motile hormogonia	Hormogonia	Hormogonia
	Light green–brown spore occasionally	Fertile trichome	Akinetes

Morphology traits were observed when isolates of each genus were growing in the solid nitrate-deficient medium.

## 4 Discussion

The current taxonomy of cyanobacteria has largely been unresolved and is still a challenge to scientists worldwide. With a valued attempt, Strunecký et al. (2023) introduced an updated classification of cyanobacteria at a family level. Therefore, a large number of cyanobacterial strains and comprehensive data on each strain are required to improve the cyanobacterial system down to the genus/species level. So far, taxonomic studies on cyanobacteria strains have generally begun by describing morphological characteristics as the first step in identifying isolated strains (Casamatta et al., 2006; Lukesova et al., 2009; Ngo et al., 2022). It is because certain morphological features play a significant role in identifying cyanobacteria in several genera rather than *Nostoc*. For the classification of nostocaceae, the molecular characterizations were considered a primary tool for the taxonomic assignment (Mateo et al., 2011; Pecundo et al., 2021; Almeida et al., 2023). According to the study of Almeida et al. (2023), phylogeny based on the 16S rRNA was proven to agree with metabolic, physiological, morphometric data, and *nifD* sequences. Therefore, we utilized the 16S rRNA gene sequence for classifying isolates. As a result, 38 isolated strains were successfully classified into three genera: *Aliinostoc*, *Aulosira*, and *Desmonostoc*, based on 16S rRNA gene sequence and phylogenetic analysis. From this point, the cyanobacteria strains within each genus serve as valuable living specimens for analyzing and exploring the signature morphological characteristics of each genus to establish new first sight markers for *Aliinostoc*, *Aulosira*, and *Desmonostoc*. The morphological characteristics can be used for rapid and preliminary identification of nostoc-like strains before applying any time-consuming technique in the laboratory.

### 4.1 Morphological markers of *Aliinostoc*

Historically, *Aliinostoc* has been a young genus of Nostocaceae, proposed by Bagchi et al. (2017); he emphasized that the important character of strains in the *Aliinostoc* clade was the presence of motile hormogonia with gas vesicles (Bagchi et al., 2017; Nowruzi and Lorenzi, 2023). In this study, we found that colonies of all the isolated *Aliinostoc* strains were grayish to brownish, sometimes gray-greenish, and never in the typical blue-green color. It was consistent with the finding of Kabirnataj et al. (2020) that the bright greenish-blue or bluish-green color occurred in the initial trichome of the *Aliinostoc magnakinetifex* SA18 and *Aliinostoc catenatum* SA24, then turned to greenish–brownish, or yellowish to brownish on prolonged growth. Merican et al. (2024) also reported that the colony color of *Aliinostoc bakau* USMNA was brownish-green to brown (Merican et al., 2024). Although the gray or brownish color has been recorded at type strain *Aliinostoc morphoplasticum* NOS (Bagchi et al., 2017) as well as *Aliinostoc vietnamicum* VP225 (Maltsevav et al., 2022), and other reported strains in this genus (Nowruzi and Lorenzi, 2023). Still, it was not noticed as a common feature of *Aliinostoc*. Nevertheless, it is interesting that the greenish-blue or greenish color of *Aliinostoc* colonies reported in the previous studies and our study was due to the occurrence of young trichomes, heterocysts, and akinetes rather than mature vegetative cells (Figures 4E,F,O). The gray or brown of mature vegetative indicated that the pigment profile of *Aliinostoc* strains contains more carotenoids and scytonemin than chlorophyll and phycocyanin (Němečková et al., 2023).

Therefore, summarizing over 18 *Aliinostoc* strains in this study and available strains from previous studies has enabled us to propose that the gray to brownish color of mature vegetative cells becomes one primary diacritical feature of the *Aliinostoc* genus in Nostocaceae. Furthermore, this study discovered a new reproduction strategy of the *Aliinostoc* genus by germinating spores (or akinetes) into short filaments inside a spherical sheath (Figure 4H). Other features of vegetative cells, heterocyst positions, or large akinetes with dense granules were congruent with *Aliinostoc* strains in the previous studies (Bagchi et al., 2017; Kabirnataj et al., 2020; Nowruzi and Lorenzi, 2023).

### 4.2 Morphological markers of *Aulosira*

*Aulosira* was initially placed in the Microchaetaceae, then was moved to Nostocaceae by Komarek and Anagnostidis (1989) and Hauer et al. (2014) and remained unclarified in identification with low sampling analysis (Casamatta et al., 2006; Ngo et al., 2022). So far, 31 species have been reported under *Aulosira* basionyms (Guiry and Guiry, 2023). Only six rRNA 16S gene sequences were available in GenBank, which left an open question about classifying most *Aulosira* species based on molecular phylogeny and led to difficulties in generalizing the signature morphological characteristics of this genus. The common features of this genus were only mentioned with firm sheath and (irregular) apoheterocytic akinete development (Komarek and Anagnostidis, 1989). In this study, seven isolated strains of the *Aulosira* genus had vegetative cells, terminal cells, intercalary heterocysts, and mucilage sheath similar to those of *A. prolifica* Bharadwaja, *A. fertilissima* var. tenuis (Anand and Rengasamy, 1978), and *Aulosira epiphytica* (Gardner) (Hindáková and Hindák, 2017). Regarding reproduction, they also formed the fertile chitromes between necrotic cells as described for *Aulosira fertilissima* (Ghose, 1924) and *Aulosira* sp. XN1103 (Ngo et al., 2022). However, these isolated strains did not resemble *Aulosira bohemensis* ISB-2 due to the absence of apoheterocytic akinete development and perpendicular hormogonia. On the other hand, they were in the clade containing the type strain *Aulosira laxa* NIES-50 but far distant from *Aulosira bohemensis* ISB-2 on the phylogenetic tree, which was also pointed out by the studies of Lukesova et al. (2009) and Hauer et al. (2014). Lukesova et al. (2009) reported that the placement of *Aulosira bohemensis* ISB-2 in the phylogenetic tree was uncertain across different phylogenetic analyses. This unsolved problem might be due to the limited taxon sampling or accurate reference sequences within the Nostocaceae at that time (even to date). Thus, Hauer et al. (2014) suggested that the *Aulosira bohemensis* ISB-2 should be revised and assigned to a new genus in the Nostocaceae at a later stage (Hauer et al., 2014).

The differentiation between the base and the apex of the filaments, with the occurrence of basal heterocysts, is due to the impact of a nitrate-deficient medium. Anand and Rengasamy (1978) reported that *A. prolifica* Bharadwaja and *A. fertilissima* var. tenuis developed filaments with purely vegetative cells in Allen and Arnon medium. The presence of basal heterocysts in short trichomes is a new diagnostic feature for the *Aulosira* phylogenetic cluster, which was previously questioned in the study by Ngo et al. (2022). Hauer et al. (2014) only noted that *Aulosira* was transferred from the Microchaetaceae to the Nostocaceae due to their isopolar and non-branching filament structure. Our results indicated that *Aulosira* trichomes were isopolar only in a nitrogen-rich medium (BG11). In nitrogen-deficient conditions (BG11_0_), they exhibited heteropolarity with basal heterocysts. The shortest filament observed in our study, which contained two vegetative cells and one heterocyst, indicated that environmental pressure for the differentiation of vegetative cells into heterocysts for atmospheric nitrogen fixation occurs early in the *Aulosira* life cycle. In other words, the presence of basal heterocysts in the BG11_0_ medium (Ngo et al., 2022) rather than in the BG11 medium of isolated *Aulosira* strains was validated in this study. The appearance of basal heterocysts made the *Aulosira* strains easily confused with *Tolypothrix* strains (Hauer et al., 2014) and *Calothrix* strains (Anahas and Muralitharan, 2015). However, it is important to emphasize that, unlike *Tolypothrix* and *Calothrix* strains, this characteristic of isolated *Aulosira* strains appeared in the short and non-tapered filaments from the initial stages of the life cycle in the nitrate-deficient medium (BG11_0_ medium).

### 4.3 Morphological markers of *Desmonostoc*

The *Desmonostoc* genus was proposed by Hrouzek et al. (2013); the arrangement of the long and parallel filaments in the microscopic colonies was described as a typical feature for members of the genus *Desmonostoc* (Hrouzek et al., 2013; de Alvarenga et al., 2018). Otherwise, there is no significant statistical difference in the shape and size of vegetative cells and heterocysts between the *Desmonostoc* and *Nostoc* strains (Hrouzek et al., 2013; Almeida et al., 2023). Among 13 *Desmonostoc* strains in our study, the strain CAVN2522 showed emerging characteristics with “compact aggregation cells enclosed in a very thick mucilaginous envelope,” which was only observed in the strain *Desmonostoc aggregatum* CF06 (Pecundo et al., 2021) and *Desmonostoc* sp. CCM-UFV069 (Almeida et al., 2023). Therefore, we suggest that the compact cells enclosed in an envelope are a rare characteristic of several *Desmonostoc* strains that only exhibit at the early life cycle stage.

Almeida et al. (2023) attempted to elucidate the morphological and phylogenetic relationships between members of the *Desmonostoc* genus and confirmed that the typical parallel trichome organization appeared in most *Desmonostoc* strains, except *Desmonostoc* sp. CCM UFV018 and CCM-UFV054 due to their phylogenetic distance from other *Desmonostoc* strains (Almeida et al., 2023). In this study, we recognized that all 13 isolated strains showed the formation of akinetes chains when growing in the BG11_0_ medium. The akinetes were round, oval, or cylindrical and successively connected in multiple directions at the intercalary or terminal of the filament. The akinete presence at mature or old filament chains in our study is congruent with observation at mid-late-stationary phases by Almeida et al. (2023). Therefore, we suggest that the presence of akinete chains with irregular directions can be one of the important diagnostic characteristics of the *Desmonostoc* genus. Nevertheless, we notice the critical role of akinetes in reproduction within the *Desmonostoc* genus by germination outside (Figure 7C) or inside (Figure 7D) the maternity filaments. Although inside germination was rarely observed in the *Desmonostoc* strains, the only record was at *Desmonostoc muscorum* NIVA-CYA 817 by Hrouzek et al. (2013); in this study, we provide further proof of akinetes germination inside the filament, both in perpendicular and in parallel division planes (Figure 7D). The variety in akinete germinations for the reproduction of *Desmonostoc* strains in the nitrate-deficient medium (BG11_0_) in this study involved their adaptation ability and thrives in the paddy soil ecosystem. The biomes of this habitat were frequently influenced by the lack of fertilizer between two cultivation seasons or farming activities.

## 5 Conclusion

Owing to the molecular technology, we have classified 26 of 38 isolated strains into the seven species within three genera (*Aliinostoc*, *Aulosira*, or *Desmonostoc*), in which the strain CAVN2522 was nomenclatured *Desmonostoc linckia* CAVN2522 after the strain *Nostoc linckia* IAM M-251 was renamed *Desmonostoc linckia* IAM M-251 by Hrouzek et al. (2013). Twelve isolated strains, including four strains belonging to the genus *Aulosira* (CAVN0801, CAVN2540, CAVN2544, and CAVN8202), one strain of the genus *Aliinostoc* (CAVN8235), and seven strains of the genus *Desmonostoc* (CAVN2403, CAVN2513, CAVN2525, CAVN2560, CAVN6515, CAVN8204, and CAVN8213), have not yet been identified to the species level due to a lack of reference sequences for type species. According to Almeida et al. (2023), the *nifH*-based phylogenetic tree may show a different topology from 16S rRNA phylogeny (Almeida et al., 2023) and is more consistent with ITS data for species-level delimitation (Genuário et al., 2015). Therefore, in the subsequent study, these unidentified species will be classified in-depth to identify novel/new species using ITS secondary structure along with *nifH* sequences and whole genome sequence. The morphology markers of *Aliinostoc*, *Aulosira*, or *Desmonostoc* genera pointed out by this study can provide a useful initial assessment for cyanobacterial classification. We also suggest that the taxonomy of cyanobacteria with *Nostoc*-like morphology should start with phylogenetic analysis primarily based on 16S rRNA gene sequences, following a profound observation of morphological features. Recently, many strains of cyanobacteria appeared in mis-nomenclature. Therefore, the data on 16S rRNA gene sequences and detailed morphological characteristics of *Aliinostoc*, *Aulosira*, and *Desmonostoc* genera in this study would be a valuable reference for cyanobacteria systematic research.

## Data Availability

Partial 16S RNA gene sequences of 38 isolates are available in the NCBI GenBank under accession numbers PQ538886–PQ538923.

## Supplementary material

SUPPLEMENTARY FIGURE 1 Phylogram derived from Maximum Likelihood (ML) analysis of the combined 16S dataset of *Aulosira*, *Aliinostoc* and *Desmonostoc*. The tree was generated using FastTree 2. Support values at nodes were based on Shimodaira and Hasegawa (1999) implemented in FastTree. Name of the isolated strains are in red.
