# Supplementary figures and images for: Classification of Nostoc-like cyanobacteria isolated from paddy soil into Aliinostoc, Aulosira, and Desmonostoc
Source: Front Microbiol. 2025 May 20;16:1581725. doi: 10.3389/fmicb.2025.1581725 (PMC12130836; doi:10.3389/fmicb.2025.1581725)

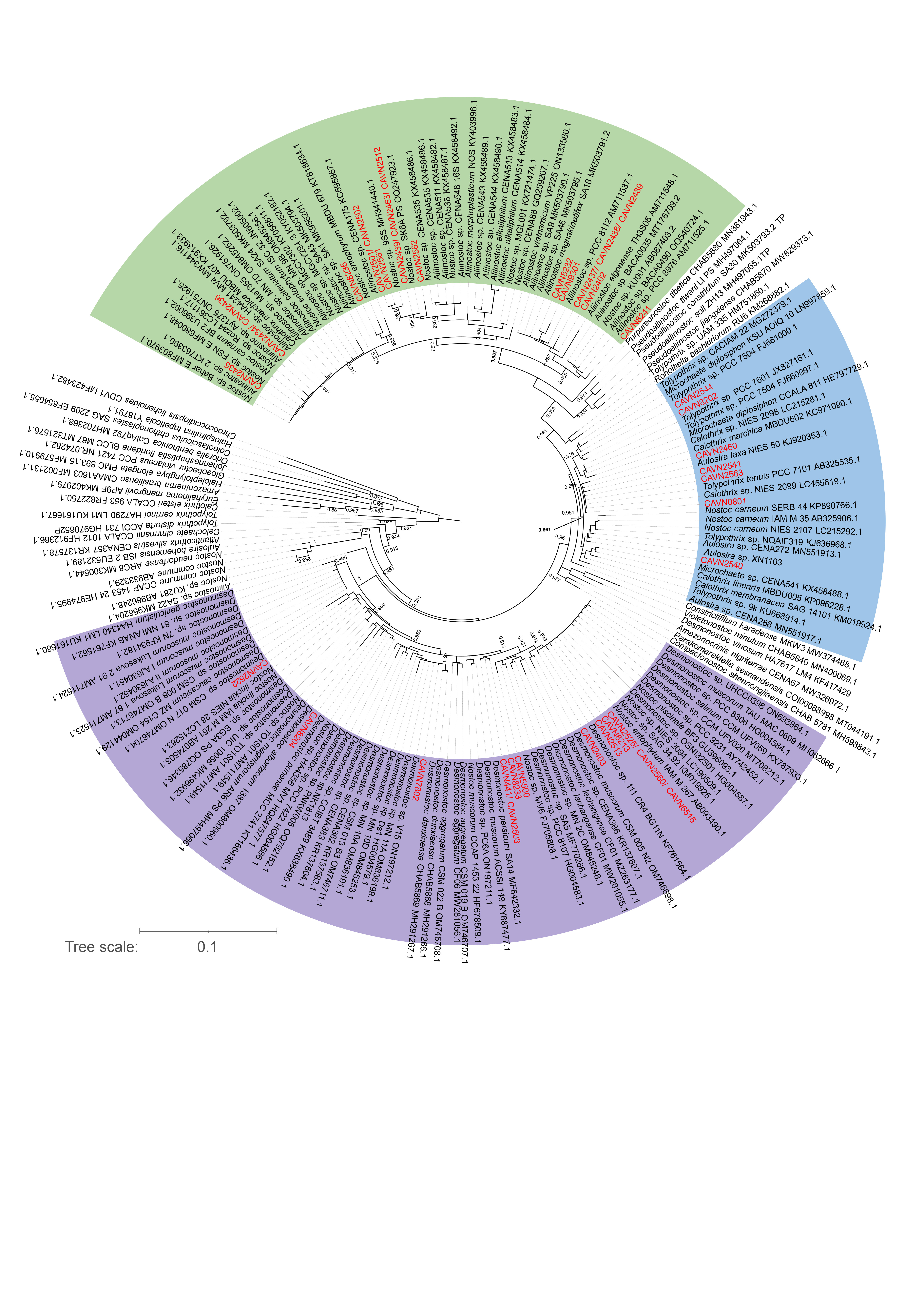

Supplement: SUPPLEMENTARY FIGURE 1 — Phylogram derived from Maximum Likelihood (ML) analysis of the combined 16S dataset of Aulosira, Aliinostoc and Desmonostoc. The tree was generated using FastTree 2. Support values at nodes were based on Shimodaira and Hasegawa (1999) implemented in FastTree. Name of the isolated strains are in red. [file Image_1.jpeg]
